# Supplementary material for: Mortality in individuals treated with COVID-19 convalescent plasma varies with the geographic provenance of donors
Source: Nat Commun. 2021 Aug 11;12:4864. doi: 10.1038/s41467-021-25113-5 (PMC8357797; doi:10.1038/s41467-021-25113-5)
Supplement: Supplementary file 1 — Supplementary Information [file 41467_2021_25113_MOESM1_ESM.pdf]

SUPPLEMENTARY INFORMATION

This supplementary document has been provided by the authors to give readers additional information about their work. Supplement to: Kunze KL, Johnson PW, van Helmond N, et al. Mortality in individuals treated with COVID-19 convalescent plasma varies with the geographic provenance of donors.

Supplementary Tables

Supplementary Table 1. Characteristics of Patients with COVID-19 who Received Convalescent Plasma, According to Donor Location

	Patients Receiving Distantly-Sourced Plasma (N=14,864)	Patients Receiving Near-Sourced Plasma (N=13,088)	All Patients (N=27,952)	Standardized Difference[†]
Age category at enrollment				0.034
18-39 years	2,466/14,864 (16.6)	2,327/13,088 (17.8)	4,793/27,952 (17.1)	
40-50 years	3,504/14,864 (23.6)	3,015/13,088 (23.0)	6,519/27,952 (23.3)	
50-60 years	5,604/14,864 (37.7)	4,828/13,088 (36.9)	10,432/27,952 (37.3)	
60-65 years	3,290/14,864 (22.1)	2,918/13,088 (22.3)	6,208/27,952 (22.2)	
Gender[a]				0.009
Women	6,146/14,864 (41.3)	5,470/13,088 (41.8)	11,616/27,952 (41.6)	
Men	8,650/14,864 (58.2)	7,559/13,088 (57.8)	16,209/27,952 (58.0)	
Other	68/14,864 (0.5)	59/13,088 (0.5)	127/27,952 (0.5)	
Weight Status[b]				0.023
Underweight or Normal Weight	1,320/14,841 (8.9)	1,226/13,085 (9.4)	2,546/27,926 (9.1)	
Overweight	3,424/14,841 (23.1)	3,029/13,085 (23.1)	6,453/27,926 (23.1)	
Class 1 Obesity	3,938/14,841 (26.5)	3,366/13,085 (25.7)	7,304/27,926 (26.2)	
Class 2 Obesity	2,595/14,841 (17.5)	2,296/13,085 (17.5)	4,891/27,926 (17.5)	
Class 3 Obesity	3,564/14,841 (24.0)	3,168/13,085 (24.2)	6,732/27,926 (24.1)	
Race[c]				**0.167**
White	7,393/14,864 (49.7)	6,300/13,088 (48.1)	13,693/27,952 (49.0)	
Black	3,347/14,864 (22.5)	2,282/13,088 (17.4)	5,629/27,952 (20.1)	
Other Race	4,124/14,864 (27.7)	4,506/13,088 (34.4)	8,630/27,952 (30.9)	
Ethnicity				0.050
Hispanic/Latino	6,727/14,864 (45.3)	5,598/13,088 (42.8)	12,325/27,952 (44.1)	
Not Hispanic/Latino	8,137/14,864 (54.7)	7,490/13,088 (57.2)	15,627/27,952 (55.9)	
US Census Region				**0.476**
Midwest	1,056/14,847 (7.1)	1,809/13,052 (13.9)	2,865/27,899 (10.3)	
Northeast	108/14,847 (0.7)	566/13,052 (4.3)	674/27,899 (2.4)	
South	11,234/14,847 (75.7)	7,132/13,052 (54.6)	18,366/27,899 (65.8)	
West	2,449/14,847 (16.5)	3,545/13,052 (27.2)	5,994/27,899 (21.5)	
Time to Infusion Category				0.073
≤ 3 days	6,888/14,864 (46.3)	6,545/13,088 (50.0)	13,433/27,952 (48.1)	
≥ 4 days	7,976/14,864 (53.7)	6,543/13,088 (50.0)	14,519/27,952 (51.9)	
Treatment Month				**0.209**
June	3,046/14,864 (20.5)	3,215/13,088 (24.6)	6,261/27,952 (22.4)	
July	7,827/14,864 (52.7)	5,532/13,088 (42.3)	13,359/27,952 (47.8)	
August	3,991/14,864 (26.9)	4,341/13,088 (33.2)	8,332/27,952 (29.8)	
Clinical Status				

Supplementary Table 1. Characteristics of Patients with COVID-19 who Received Convalescent Plasma, According to Donor Location

	Patients Receiving Distantly-Sourced Plasma (N=14,864)	Patients Receiving Near-Sourced Plasma (N=13,088)	All Patients (N=27,952)	Standardized Difference[†]
ICU care before infusion	3,783/14,864 (25.5)	3,590/13,088 (27.4)	7,373/27,952 (26.4)	0.045
Severe/life-threatening COVID-19	8,144/14,864 (54.8)	7,048/13,088 (53.9)	15,192/27,952 (54.4)	0.019
Risk factors for severe Covid-19 in subgroup of patients with severe or life-threatening Covid-19				
Respiratory failure	4,107/8,144 (50.4)	3,124/7,048 (44.3)	7,231/15,192 (47.6)	**0.123**
Dyspnea	6,812/8,144 (83.6)	6,101/7,048 (86.6)	12,913/15,192 (85.0)	0.082
Blood oxygen saturation ≤ 93%	6,457/8,144 (79.3)	5,883/7,048 (83.5)	12,340/15,192 (81.2)	**0.108**
Lung infiltrates > 50% within 24 to 48 hours	2,619/8,144 (32.2)	2,455/7,048 (34.8)	5,074/15,192 (33.4)	0.057
Respiratory frequency ≥ 30/min	2,975/8,144 (36.5)	2,800/7,048 (39.7)	5,775/15,192 (38.0)	0.066
PaO2:FiO2 ratio < 300[d]	1,373/8,144 (16.9)	1,290/7,048 (18.3)	2,663/15,192 (17.5)	0.038
Multiple organ dysfunction or failure	242/8,144 (3.0)	185/7,048 (2.6)	427/15,192 (2.8)	0.021
Septic shock	118/8,144 (1.4)	93/7,048 (1.3)	211/15,192 (1.4)	0.011
Medications received during hospital stay				
ARB[e]	324/4,910 (6.6)	230/4,386 (5.2)	554/9,296 (6.0)	0.057
ACE inhibitors[f]	386/4,910 (7.9)	359/4,386 (8.2)	745/9,296 (8.0)	0.012
Azithromycin	2,553/4,910 (52.0)	2,027/4,386 (46.2)	4,580/9,296 (49.3)	**0.116**
Remdesivir	2,472/4,910 (50.3)	2,355/4,386 (53.7)	4,827/9,296 (51.9)	0.067
Steroids	3,934/4,910 (80.1)	3,043/4,386 (69.4)	6,977/9,296 (75.1)	**0.249**
(Hydroxy)chloroquine	126/4,910 (2.6)	94/4,386 (2.1)	220/9,296 (2.4)	0.028

All values are represented as number/total number (percent).

[†]Standardized Difference = difference in proportions divided by standard error; imbalance is defined as an absolute value greater than 0.1 and are shown in bold

[a]In the “Other” gender category, 51 patients were intersex, 46 were transgender, and 30 chose not to disclose.

[b]Weight Status based on BMI. Underweight or Normal Weight: Below 25; Overweight: 25 - 29; Class 1 Obesity: 30 - 34; Class 2 Obesity: 35 - 39; Class 3 Obesity: 40+

[c]In the “Other race” category, 463 patients were American Indian/Alaska Native, 223 were Native Hawaiian/Other Pacific Islander, 107 were multiracial, and 7,108 were reported as other or unknown.

[d]PaO2:FiO2 = Ratio of partial pressure of arterial oxygen to fraction of inspired oxygen

[e]ARB = Angiotensin II receptor blockers

[f]ACE = Angiotensin-converting-enzyme

Supplementary Table 2. Crude mortality rates by demographics and donor proximity

	Patients Receiving Distantly-Sourced Plasma (N = 14,864)	Patients Receiving Near-Sourced Plasma (N = 13,088)	All patients (N = 27,952)
Gender			
Women	9.78% (9.06%, 10.55%)	6.82% (6.18%, 7.52%)	8.38% (7.89%, 8.90%)
Men	11.46% (10.80%, 12.15%)	9.91% (9.26%, 10.60%)	10.73% (10.27%, 11.22%)
Other	16.18% (9.28%, 26.69%)	5.08% (1.74%, 13.92%)	11.02% (6.68%, 17.65%)
Age			
18 to 39 y	3.44% (2.79%, 4.24%)	2.87% (2.26%, 3.63%)	3.16% (2.70%, 3.70%)
40 to 59 y	9.85% (9.25%, 10.48%)	7.78% (7.21%, 8.40%)	8.89% (8.47%, 9.33%)
60 to 65 y	18.66% (17.38%, 20.02%)	15.15% (13.91%, 16.49%)	17.01% (16.11%, 17.96%)
Race			
White	10.88% (10.19%, 11.61%)	8.54% (7.87%, 9.26%)	9.80% (9.31%, 10.31%)
Black	10.85% (9.84%, 11.94%)	9.42% (8.29%, 10.69%)	10.27% (9.50%, 11.09%)
Other race	10.57% (9.67%, 11.55%)	8.26% (7.49%, 9.10%)	9.36% (8.77%, 10.00%)
Ethnicity			
Hispanic/Latino	11.71% (10.97%, 12.50%)	8.61% (7.90%, 9.37%)	10.30% (9.78%, 10.85%)
Not Hispanic/Latino	10.02% (9.38%, 10.69%)	8.58% (7.97%, 9.24%)	9.33% (8.88%, 9.80%)
Treatment Region			
Midwest	6.06% (4.77%, 7.67%)	5.53% (4.57%, 6.68%)	5.72% (4.93%, 6.64%)
Northeast	6.48% (3.17%, 12.78%)	7.77% (5.84%, 10.28%)	7.57% (5.80%, 9.81%)
South	11.16% (10.59%, 11.76%)	8.96% (8.32%, 9.64%)	10.31% (9.88%, 10.76%)
West	11.35% (10.16%, 12.67%)	9.53% (8.61%, 10.55%)	10.28% (9.53%, 11.07%)
Treatment Month			
June	9.29% (8.31%, 10.37%)	7.68% (6.81%, 8.65%)	8.47% (7.80%, 9.18%)
July	11.98% (11.28%, 12.72%)	9.45% (8.71%, 10.25%)	10.94% (10.42%, 11.48%)
August	9.57% (8.70%, 10.52%)	8.18% (7.40%, 9.03%)	8.85% (8.25%, 9.47%)
Plasma Donation Region			
Midwest	8.70% (7.49%, 10.09%)	5.57% (4.61%, 6.71%)	7.12% (6.33%, 8.00%)
Northeast	10.53% (9.87%, 11.24%)	7.28% (5.60%, 9.43%)	10.26% (9.63%, 10.92%)
South	11.46% (10.53%, 12.46%)	9.03% (8.38%, 9.73%)	9.94% (9.40%, 10.51%)
West	13.42% (11.54%, 15.56%)	9.56% (8.63%, 10.57%)	10.48% (9.63%, 11.40%)

Supplementary Table 3. Models of the Association between Donor Proximity and the Risk of Death

	Estimated Relative Risk (95% CI)	P value
Base Model (n = 27,952)		
Donor Distance (Near-sourced)	0.80 (0.74, 0.86)	**<0.001**
Model 2 (n = 27,899)		
Age	1.06 (1.05, 1.06)	**<0.001**
ICU care before to infusion (Yes)	2.09 (1.94, 2.25)	**<0.001**
Respiratory failure (Yes)	1.69 (1.57, 1.82)	**<0.001**
Region (Northeast)	1.47 (1.10, 1.98)	**0.010**
Region (South)	1.88 (1.61, 2.18)	**<0.001**
Region (West)	2.06 (1.75, 2.43)	**<0.001**
Gender (Men)	1.21 (1.13, 1.30)	**<0.001**
Gender (Other)	1.28 (0.78, 2.09)	0.32
Time to transfusion (days)	1.01 (1.00, 1.02)	**0.006**
Donor Distance (Near-sourced)	0.83 (0.78, 0.90)	**<0.001**
Model 3 (n = 9,279)[a]		
Age	1.06 (1.05, 1.07)	**<0.001**
ICU care before to infusion (Yes)	2.05 (1.80, 2.33)	**<0.001**
Region (Northeast)	1.83 (1.14, 2.93)	**0.012**
Region (South)	1.81 (1.36, 2.41)	**<0.001**
Region (West)	2.19 (1.63, 2.95)	**<0.001**
Weight Status (Overweight)	0.73 (0.59, 0.91)	**0.005**
Weight Status (Class 1 Obesity)	0.85 (0.69, 1.05)	0.14
Weight Status (Class 2 Obesity)	0.89 (0.71, 1.12)	0.32
Weight Status (Class 3 Obesity)	1.13 (0.91, 1.40)	0.25
Gender (Men)	1.11 (0.98, 1.26)	0.11
Gender (Other)	0.99 (0.38, 2.58)	0.99
Lung infiltrates or low PAO2:FIO2 (Yes)	1.42 (1.24, 1.62)	**<0.001**
Remdesivir (Yes)	0.81 (0.72, 0.92)	**<0.001**
Steroids (Yes)	1.46 (1.23, 1.72)	**<0.001**
Time to transfusion (days)	1.01 (0.99, 1.02)	0.25
Donor Distance (Near-sourced)	0.77 (0.68, 0.87)	**<0.001**
Model 4 (n = 27,952)[b]		
Donor Distance (Near-sourced)	0.85 (0.79, 0.92)	**<0.001**

Relative risk regression models were constructed using a generalized linear model framework. A log link and the robust variance estimator (Poisson distribution) were included to correct for the misspecified variance structure.
Donor distance was dichotomized into near-sourced (≤ 150 miles) and distantly-sourced (> 150 miles).
Region refers to the region of treatment for the infused patient.
[a]Model 3 is noticeably smaller as fewer patients had medications reported during the study time window.
[b]Model 4 is weighted based on propensity scores calculated using a gradient boosting machine. Matching was based on age, gender, race, US census region, ICU care prior to infusion, weight status, treatment month, respiratory failure, respiratory frequency ≥ 30/min, multiple organ dysfunction or failure, PaO2:FiO2 ratio < 300, blood oxygen saturation ≤ 93%, and treatment with steroids and azithromycin.

Supplementary Figures

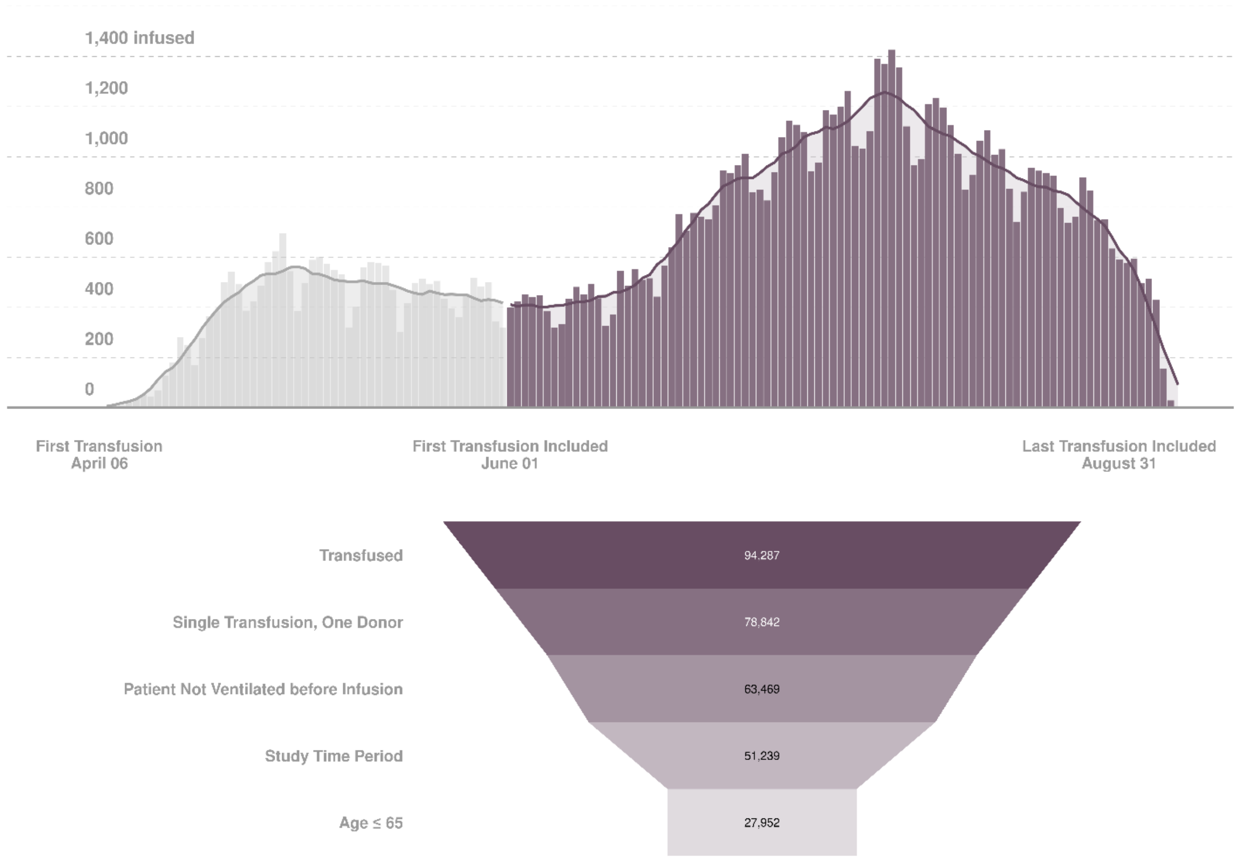

Supplementary Figure 1. Transfusions in the Expanded Access Program (EAP) for Convalescent Plasma over the Entire EAP Duration and for the Data Used in Current Study. The purple bars represent daily transfusions of new patients during the study period (June 1, 2020 to August 31, 2020), and grey bars are transfusions that occurred beforehand. The overlayed trend line represents a 7-day rolling average. The funnel diagram depicts the cohort selection criteria that resulted in the inclusion of 27,952 patients. A single transfusion from one donor may include one or two units of plasma. Color is used to highlight cohort analyzed and to show changes in cohort size during the cohort selection process.

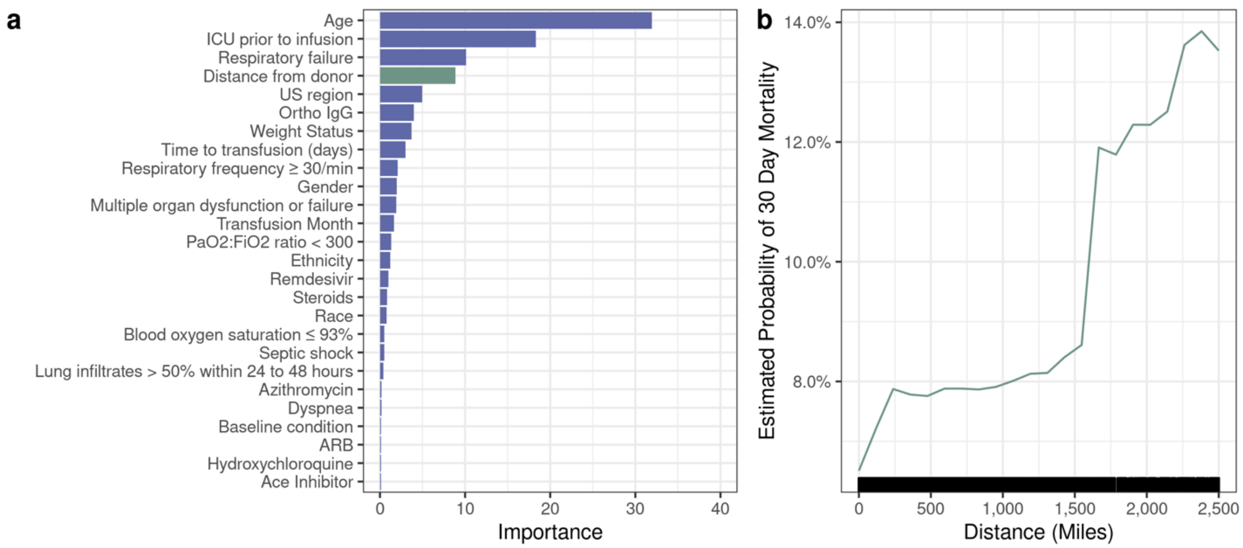

Supplementary Figure 2. The Variable Importance Plot (a) and the Partial Dependence Plot (b) from the Gradient-Boosting Machine. The variable importance plot shows the relative importance of each variable in predicting 30-day mortality. All variables included in the gradient-boosting machine are shown below. The partial dependence plot shows the estimated probability of death across donor-patient distances when accounting for the average effect of all other predictors in the model. Distance was winsorized in Figure S2b at 2500 miles. Color is used to highlight distance from donor.
