# Supplementary material for: Mortality in individuals treated with COVID-19 convalescent plasma varies with the geographic provenance of donors
Source: Nat Commun. 2021 Aug 11;12:4864. doi: 10.1038/s41467-021-25113-5 (PMC8357797; doi:10.1038/s41467-021-25113-5)
Supplement: Supplementary file 3 — Reporting Summary [file 41467_2021_25113_MOESM3_ESM.pdf]

nature research

Corresponding author(s): Michael Joyner

Last updated by author(s): Jun 30, 2021

Reporting Summary

Nature Research wishes to improve the reproducibility of the work that we publish. This form provides structure for consistency and transparency in reporting. For further information on Nature Research policies, see our Editorial Policies and the Editorial Policy Checklist.

Statistics

For all statistical analyses, confirm that the following items are present in the figure legend, table legend, main text, or Methods section.

n/a	Confirmed	
☐	☒	The exact sample size (n) for each experimental group/condition, given as a discrete number and unit of measurement
☒	☐	A statement on whether measurements were taken from distinct samples or whether the same sample was measured repeatedly
☐	☒	The statistical test(s) used AND whether they are one- or two-sided<br>*Only common tests should be described solely by name; describe more complex techniques in the Methods section.*
☐	☒	A description of all covariates tested
☒	☐	A description of any assumptions or corrections, such as tests of normality and adjustment for multiple comparisons
☐	☒	A full description of the statistical parameters including central tendency (e.g. means) or other basic estimates (e.g. regression coefficient) AND variation (e.g. standard deviation) or associated estimates of uncertainty (e.g. confidence intervals)
☐	☒	For null hypothesis testing, the test statistic (e.g. F, t, r) with confidence intervals, effect sizes, degrees of freedom and P value noted<br>*Give P values as exact values whenever suitable.*
☒	☐	For Bayesian analysis, information on the choice of priors and Markov chain Monte Carlo settings
☒	☐	For hierarchical and complex designs, identification of the appropriate level for tests and full reporting of outcomes
☒	☐	Estimates of effect sizes (e.g. Cohen's d, Pearson's r), indicating how they were calculated

Our web collection on statistics for biologists contains articles on many of the points above.

Software and code

Policy information about availability of computer code

Data collection	Patient data were collected using REDCap version 10.6.1 1.
Data analysis	Analyses were conducted using R 3.6.2. Specific R packages and versions can be provided upon request.

For manuscripts utilizing custom algorithms or software that are central to the research but not yet described in published literature, software must be made available to editors and reviewers. We strongly encourage code deposition in a community repository (e.g. GitHub). See the Nature Research guidelines for submitting code & software for further information.

Data

Policy information about availability of data

All manuscripts must include a data availability statement. This statement should provide the following information, where applicable:

- Accession codes, unique identifiers, or web links for publicly available datasets
- A list of figures that have associated raw data
- A description of any restrictions on data availability

Plasma donor neutralizing antibody data were stored in a on-premises DB 2 SQL database.Data availability statement: Study data cannot be shared publicly because of Institutional Review Board restrictions. Individual participant data underlying the results reported in this publication, along with a data dictionary, may be made available to approved investigators for secondary analyses following the completion of the objectives of the United States Expanded Access Program to COVID-19 convalescent plasma. Limited and de-identified data sets will be deposited into a research data repository and may be shared with investigators under controlled access procedures as approved by the Mayo Clinic Institutional Review Board. A scientific committee will review requests for the conduct of protocols approved or determined to be exempt by an Institutional Review Board. Requestors may be required to sign a data use agreement. Data sharing must be compliant with all applicable Mayo Clinic policies. Interested parties may contact the Mayo Clinic Institutional Review Board at uscovidplasma@mayo.edu.

Field-specific reporting

Please select the one below that is the best fit for your research. If you are not sure, read the appropriate sections before making your selection.

- [x] Life sciences
- [ ] Behavioural & social sciences
- [ ] Ecological, evolutionary & environmental sciences

For a reference copy of the document with all sections, see nature.com/documents/nr-reporting-summary-flat.pdf

Life sciences study design

All studies must disclose on these points even when the disclosure is negative.

Sample size	Data from all available patients who met inclusion criteria were analyzed in this study. The final sample size included 27,952 patients, and a near-equal distribution of near-sourced and distantly-sourced plasma was observed in this final dataset.
Data exclusions	Hospitalized patients with COVID-19 who enrolled in the Expanded Access Program (EAP) for convalescent plasma, were between the ages of 18 and 65 years old, and were transfused with one or two units of convalescent plasma from a single donor between June 1, 2020 and August 31, 2020 were included in this analysis. Mechanically ventilated patients were excluded because current evidence suggests that convalescent plasma is not effective in this subpopulation. Given that age is a pronounced risk factor for mortality, patients over age 65 were excluded to further explore the impacts of other potential risk factors. See Supplementary Figure 1.
Replication	This is a retrospective study of an existing dataset collected during the COVID-19 pandemic. All final datasets were frozen, and implemented code has been version controlled via git.
Randomization	Randomizationwasnotimplemented;however,thelargesamplesizeallowedustocontrolforpotentialconfoundersthroughsubgroupanalysis (e.g.,Cochran-Mantel-Haenszeltechniques).
Blinding	Blinding was not conducted or relevant to this study.TheEAPwasdesignedtotreatallhospitalizedpatientswithsevereorlife-threateningCOVID-19.

Reporting for specific materials, systems and methods

We require information from authors about some types of materials, experimental systems and methods used in many studies. Here, indicate whether each material, system or method listed is relevant to your study. If you are not sure if a list item applies to your research, read the appropriate section before selecting a response.

Materials & experimental systems

n/a	Involved in the study	
x		Antibodies
x		Eukaryotic cell lines
x		Palaeontology and archaeology
x		Animals and other organisms
	x	Human research participants
	x	Clinical data
x		Dual use research of concern

Methods

n/a	Involved in the study	
x		ChIP-seq
x		Flow cytometry
x		MRI-based neuroimaging

Human research participants

Policy information about studies involving human research participants

Population characteristics	Patients were not selected based on their characteristics. The EAP was designed to treat hospitalized patients with severe orlife-threatening COVID-19 regardless of patient's age, gender, or other characteristics. See Supplementary Table 1 for allrecorded characteristics of patients included in this analysis.
Recruitment	Physicians enrolled patients at participating hospitals via the study website after themselves enrolling in the EAP as a participating physician. All physicians licensed in the U.S. were allowed to enroll patients and acquire convalescent plasma for infusion. This method of enrollment would seem to make self-selection to receive plasma unlikely. This was not a study with selected sites where eligible patients could seek out a participating hospital to increase their chance to receive plasma. A potential bias may be that physicians perhaps reserved plasma, being an experimental therapy, for their more severe patients. This would seem to be supported by the relatively high rate of ICU admission and other indicators of severity in our cohort. That selection bias however would seem to be unlikely to impact the efficacy of near-sourced vs. distantly sourced plasma.
Ethics oversight	The study was approved by the Mayo Clinic Institutional Review Board (IND 19832 Sponsor: Dr. Michael J. Joyner, MD) andcontinuously monitored by Mayo Clinic's Data and Safety Monitoring Board.

Note that full information on the approval of the study protocol must also be provided in the manuscript.

Clinical data

Policy information about clinical studies

All manuscripts should comply with the ICMJE guidelines for publication of clinical research and a completed CONSORT checklist must be included with all submissions.

Clinical trial registration	ClinicalTrials.gov number, NCT04338360.
Study protocol	All versions of the full trial protocol can be accessed at uscovidplasma.com
Data collection	The centralized national registry was housed on servers at Mayo Clinic. Data collection occurred at 2,722 acute care facilities and hospitals across the US. Patients were enrolled in the EAP between April 1st, 2020 and August 31st, 2020. Data collection ended on December 15th, 2020. Only patients enrolled on or after June 1st were included in this analysis.
Outcomes	Delineated in section 3 of the protocol. Primary outcome: assess the availability of convalescent plasma assessed via number of convalescent plasma transfusions and standardized blood product identifier (ISBT 128 code). Secondary outcome: serious adverse events assessed via registry reporting tools and adjudicated in real-time by team of trained physicians from Mayo Clinic. Tertiary outcomes: length of stay in hospital and ICU, and survival.
